# The relationship between political affiliation and beliefs about sources of “fake news”

**DOI:** 10.1186/s41235-021-00278-1

**Published:** 2021-02-12

**Authors:** Robert B. Michael, Brooke O. Breaux

**Affiliations:** Department of Psychology, University of Louisiana at Lafayette, PO Box 43644, Lafayette, LA 70504-3644 USA

**Keywords:** Desirability bias, Fake news, Journalism, Politics

## Abstract

The 2016 US Presidential campaign saw an explosion in popularity for the term “fake news.” This phenomenon raises interesting questions: Which news sources do people believe are fake, and what do people think “fake news” means? One possibility is that beliefs about the news reflect a bias to disbelieve information that conflicts with existing beliefs and desires. If so, then news sources people consider “fake” might differ according to political affiliation. To test this idea, we asked people to tell us what “fake news” means, and to rate several news sources for the extent to which each provides real news, fake news, and propaganda. We found that political affiliation influenced people’s descriptions and their beliefs about which news sources are “fake.” These results have implications for people’s interpretations of news information and for the extent to which people can be misled by factually incorrect journalism.

## Significance statement

Advances in technology have made it easier than ever for nefarious groups to launch and co-ordinate disinformation campaigns. Concerns about the manipulation of popular social media websites like Facebook, Twitter, and Reddit dovetail with the relatively recent explosive rise in popularity of the term “fake news.” People are faced with an increasingly difficult problem of sorting fact from fiction. How do people decide what news to believe? We suspected that the news sources people trust are the ones that confirm their pre-existing beliefs, and the news sources people distrust are the ones that conflict with their pre-existing beliefs. We asked people to rate a variety of news sources according to how “real” or “fake” they were and found differing patterns of beliefs across the political spectrum. Our results suggest that political affiliation might drive skepticism—or the lack thereof—of news information.

“You are fake news.” — Donald J. Trump, 45^th^ President of the United States of America.

**Fig. 1:**
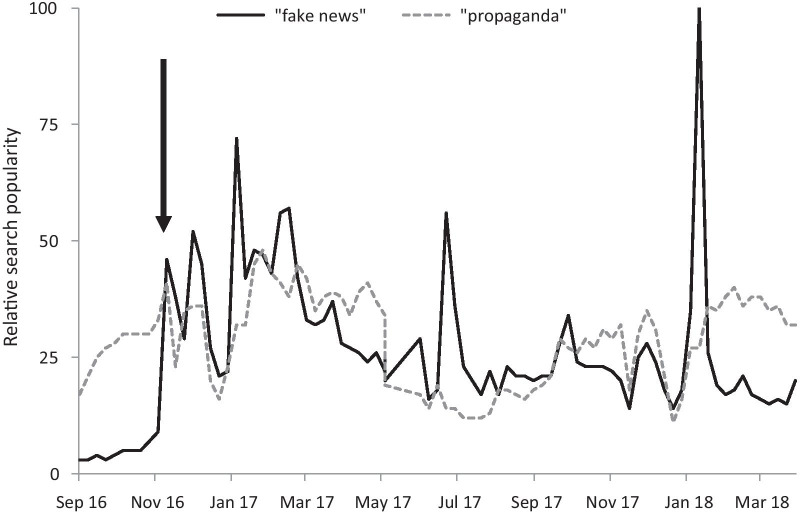
Google Trends data for the search terms “fake news” and “propaganda.” Searches for “fake news” prior to September 2016 were virtually non-existent. The arrow highlights the first spike in search popularity around the 2016 US Presidential election

The phrase “fake news” took center stage during the 2016 US Presidential election. Figure 1 displays data from Google Trends—a public web service showing the relative frequency of search terms—and highlights the rise in popularity of the phrase (Google Trends 2018). As the figure shows, searches for “fake news” were almost unheard of in September 2016. But searches increased as the election drew near and skyrocketed after the election in mid-January 2017. This peak was the result of then president-elect Donald J. Trump’s denouncement of CNN as fake news, during his first press conference on the 11th of January (Savransky 2017).

Fake news quickly became a worrying phenomenon. Multiple groups sprouted efforts to educate the public in sorting fact from fiction, including: The News Literacy Project (http://www.thenewsliteracyproject.org), the Washington Post (Berinsky 2017), and even social media giant Facebook (Price 2017). Social scientists have joined these efforts too. Recent evidence, for example, shows that deliberately generating misleading information in the guise of a game improves the ability to detect and resist fake news (Roozenbeek and van der Linden 2019). Other recent work shows that people are better able to remember corrections to false statements when given reminders of those statements (Wahlheim et al. 2020).

Ultimately, it is difficult to know how successful such attempts will be, but there is reason to believe we need them. Misinformation researchers have repeatedly shown that people fail to remember retractions of false or misleading information, especially with the passage of time (Lewandowsky et al. 2012; Rich and Zaragoza 2020). Or take the now-debunked Pizzagate conspiracy theory, which came to a dramatic conclusion when a man fired three rifle shots in a pizzeria because he believed that the restaurant was involved in a child-sex ring linked to members of the Democratic party. Pizzagate was born from fake news: The roots of the conspiracy theory trace back to a white-supremacist’s twitter post that went viral (Akpan 2016).

Another word for a similar phenomenon to “fake news” is already part of our vocabulary: propaganda. The rise of the phrase “fake news” as an alternative label for what might at times be considered propaganda is politically and psychologically intriguing, and leads to interesting questions: Which news sources do people consider real news, or fake news, and why? Do the news sources people categorize as fake news differ from those they categorize as propaganda? One possibility is that people interpret the phrase “fake news” to simply mean a new way of saying “propaganda.” But an alternative possibility is that people make distinctions between fake news and propaganda. For example, satirical sources of information like The Onion might reasonably be classified as fake news, but not necessarily propaganda (Tandoc et al. 2018).

The answers to these questions could inform theories of persuasion and reasoning that explain how people interpret information—including information reported by the media. One such theory proposes that the more involved people are with a topic, the more likely they are to attend to the content of that message over less central information, like the credibility of the source (Greenwald 1968; Petty and Cacioppo 1981). An alternative instead proposes that the more involved people are with a topic, the narrower the range of ideas they will find acceptable (Sherif et al. 1965). One prominent theory that speaks closely to the issue of “fake news” suggests that people’s motivations—their preference for some outcome—affect the strategies used when reasoning (Epley and Gilovich 2016; Kunda 1990). More specifically, this theory explains how our goals can steer information processing away from rationality and accuracy, leading to biased reasoning. In addition, this theory helps explain how and under what conditions people are likely to form partisan beliefs (Bolsen et al. 2014; Pennycook and Rand 2019). Taken together, these theories are informative when considering important issues, such as people’s trust or distrust of the media.

Several factors predict how strongly people distrust the media, including: extremity of attitudes, political partisanship, political ideology, trust in the government, and economic beliefs (Gunther 1988; Jones 2004; Lee 2010). More specifically, we know that people with particularly strong positions on topics, people who identify as “strong republicans” or “strong conservatives,” and people who report low trust in the government are the most likely to claim they almost never trust the media (Gunther 1988; Jones 2004). In addition, a pessimistic view of the economy predicts political distrust, which in turn predicts distrust of the media (Lee 2010). This distrust influences what news people ultimately believe and how they behave. Research shows, for example, that while fake news is relatively uncommon, it is heavily concentrated among conservatives, who—along with the elderly—are the most likely to spread such news (Grinberg et al. 2019; Guess et al. 2019). And during a global pandemic, distrust in media accuracy among conservatives has led to misperceptions of risk and non-compliance with behaviors that mitigate the spread of COVID-19 (Rothgerber et al. 2020).

There are therefore reasons to suspect that people’s political affiliation could determine which news sources they consider fake news. Related research supports this prediction. We know, for example, that conservatism is associated with the tendency to find harmful but false information credible (Fessler et al. 2017). That finding is consistent with work showing that conservatism is associated with sensitivity to threat (Lilienfeld and Latzman 2014). We also know that people are biased toward processing information confirming pre-existing beliefs and desires and biased away from processing ideologically challenging information (Collins et al. 2017; Nickerson 1998; Tappin et al. 2017). Moreover, these findings are not merely academic: An NPR-Ipsos poll showed that people’s preferred sources of news influence their feelings on immigration (Rose 2018).

But taken together, these data and theoretical accounts tell us only how the media is perceived in general. Answers to questions about which specific news sources people categorize as real or fake news, and why, would add nuance to these accounts. Moreover, the answers to these questions could invigorate research. One relevant avenue of interest to memory researchers, for example, relates to how effectively people can distinguish between remembered information that came from a “real” source versus that which came from a “fake” one (Johnson et al. 1993). More specifically, we know from the literature that people are more likely to misremember fake political news as real news when the content is consistent with people’s pre-existing beliefs (Frenda et al. 2013; Murphy et al. 2019). Recent evidence suggests that source information could influence these distortions, making implausible information seem plausible, and vice versa (Dias et al. 2020).

Based on this body of work, we might anticipate that the news sources conservatives classify as fake news will be distinct from the news sources liberals classify as fake news. Some recent evidence provides support for this idea, showing partisan differences in what springs to mind when encountering the term “fake news” (van der Linden et al. 2020). We also know, however, that people from opposing sides of the political spectrum can paradoxically both view the same news information as biased against their side (Perloff 2015). We might expect, then, that people outside of the political center are most likely to classify news sources in general as fake news.

In this article, we report a series of experiments assessing people’s beliefs regarding “fake news.” More specifically, we ask three key questions. First, how does political affiliation influence the extent to which people believe various news sources report real news, fake news, and propaganda? Second, to what extent does political affiliation affect how people interpret the term “fake news”? Third, how are these beliefs and interpretations changing over time? To answer the first question, we asked people to rate the extent to which several news sources provide real news, fake news, and propaganda. We also asked people to self-report their political affiliation. Based on the literature, we hypothesized that people’s political motivations would lead to reasoning strategies focused on agreement with pre-existing beliefs. We therefore predicted that news sources given high ratings by people who identify left would be given low ratings by people who identify right—and vice versa. To answer the second question, we asked people to tell us what the terms “fake news” and “propaganda” mean to them, and then looked to see how people’s responses differed according to their political affiliation. To answer the third question, we repeated this procedure across three time points: March 2017, April 2018, and August 2020.

## Experiment 1

Our first experiment was not pre-registered, and we therefore encourage cautious interpretation of the results. The remaining two experiments were pre-registered, and all experiments followed the same basic method. Data are available from https://osf.io/x7jnu.

### Method

#### Subjects

Across all experiments, we aimed to recruit as many subjects as possible, based on funding availability. No subject participated in more than one experiment. This goal resulted in a target sample size of 200 subjects for this experiment. Ultimately, we recruited a total of 203 Amazon Mechanical Turk workers who live in the USA, because Mechanical Turk and Qualtrics—our experimental software—interact such that it is possible to unintentionally collect more data points than requested (90 women, 113 men, *M*_age_ = 36 years, age range: 19–72 years). According to a sensitivity analysis, this sample size gives us adequate power to detect a small interaction effect by conventional standards (*f* = 0.06).

#### Design

We manipulated News Source within subjects. In addition, subjects were assigned into one of three Political Identification groups based on responses to a political identification question.

#### Materials and procedure

Subjects first answered age and sex demographic questions. Next, subjects rated the news sources. We constructed the list of sources as follows. First, we decided that the list should span the political spectrum and vary in terms of journalistic integrity. We then gathered a list of popular news sites according to Amazon’s Alexa Internet (Alexa Internet 2018) and the Pew Research Center (Olmstead et al. 2011). Next, we added an additional eight news sources known for sensationalist reporting. Finally, the first author provided the list of sources to his research laboratory for discussion. There was agreement that the list featured a mix of sources spanning the political spectrum and varying in journalistic integrity. Table 1 presents the final list of 42 news sources.

**Table 1:** List of news sources

Name	Website
ABC News	www.abcnews.go.com
Addicting Info	www.addictinginfo.org/category/news/
Al Jazeera	www.aljazeera.com/news/
AOL	www.aol.com/news/
Associated Press	www.ap.org/en-us/
Atlantic	www.theatlantic.com
BBC	www.bbc.com/news/
Boston Globe	www.bostonglobe.com
Breitbart	www.breitbart.com
CBS News	www.cbsnews.com
Chicago Tribune	www.chicagotribune.com
CNN	www.cnn.com
Daily Caller	www.dailycaller.com
Daily Mail	www.dailymail.co.uk/ushome/index.shtml
David Avocado Wolfe	www.davidwolfe.com
Drudge Report	www.drudgereport.com
Economist	www.economist.com
Fox News	www.foxnews.com
Google News	www.news.google.com
Guardian	www.theguardian.com/us/
Huffington Post	www.huffingtonpost.com
Infowars	www.infowars.com
Intercept	www.theintercept.com
Los Angeles Times	www.latimes.com
MSNBC	www.msnbc.com
NPR	www.npr.org/sections/news/
New York Daily News	www.nydailynews.com
New York Post	www.nypost.com
New York Times	www.nytimes.com
Occupy Democrats	www.occupydemocrats.com
Red State	www.redstate.com
Reuters	www.reuters.com
Russia Today	www.rt.com/news/
San Francisco Chronicle	www.sfchronicle.com
Slate	www.slate.com
The Blaze	www.theblaze.com/news/
USA Today	www.usatoday.com
US Uncut	www.usuncut.com
Vox	www.vox.com
Wall Street Journal	www.wsj.com
Washington Post	www.washingtonpost.com
Yahoo	www.yahoo.com/news/

List of news sources and associated websites

Subjects made 3 ratings for each source. We randomized the order of sources for each subject and each source appeared on its own page. Before the rating task began, we told subjects: “For each news source, we would like you to tell us how much you believe each is a source of real news, fake news, and propaganda. These three categories are not mutually exclusive. For example, a news source might report some real news, but it might report some fake news too.” To encourage honest responding, we also told subjects that there were no right or wrong answers and that we were interested only in what they thought. For each source, subjects saw the name of the source (e.g., “The New York Times”) above three 5-point Likert scales, labeled “Real news,” “Fake news,” and “Propaganda.” Subjects rated each source using these three scales (1 = *Definitely not*; 2 = *Probably not*; 3 = *Might or might not be*; 4 = *Probably is*; 5 = *Definitely is*).

Subjects then answered four additional questions. First, we asked subjects how much time on average they devoted to news each day, using a 4-point scale (1 = *Fewer than 30 min*; 2 = *Between 30 min and 1 h*; 3 = *Between 1 and 2 h*; 4 = *More than 2 h*).^1^ Second, we asked subjects their political identification, using a 7-point scale (1 = *Far left*; 2 = *Middle left*; 3 = *Weak left*; 4 = *Center*; 5 = *Weak right*; 6 = *Middle right*; 7 = *Far right*). Third, we asked subjects: “Consider the terms ‘fake news’ and ‘propaganda.’ What do these terms mean to you? How are they similar and different?” Finally, we asked subjects what they thought the study was about.

### Results and discussion

#### Beliefs about news sources

We first examined the extent to which the ratings of real news, fake news, and propaganda were related to each other, collapsed across news sources. More specifically, we calculated the average of each subject’s 42 real news ratings, 42 fake news ratings, and 42 propaganda ratings. Table 2 presents the Pearson correlations for these three measures and their associated 95% confidence intervals (CIs). As the table shows, real news ratings were strongly and negatively associated with fake news ratings and propaganda ratings, and fake news ratings were strongly and positively associated with propaganda ratings. These data suggest—at least for the list we used—that news agencies rated highly as sources of real news are unlikely to be rated highly as sources of fake news or propaganda, and that news agencies rated highly as sources of fake news are likely to be rated highly as sources of propaganda.

**Table 2:** Averaged real news, fake news, and propaganda rating correlations

	Fake news	Propaganda
2017		
Real news	− .69 [− .76, − .62]	− .52 [− .62, − .41]
Fake news		.79 [.73, .84]
2018		
Real news	− .33 [− .45, − .20]	− .26 [− .38, − .12]
Fake news		.80 [.74, .84]
2020		
Real news	.16 [.04, .27]	.39 [.29, .48]
Fake news		.75 [.70, .80]

Pearson correlations and associated 95% confidence intervals for averaged real news, fake news, and propaganda ratings. 2017 data are from Experiment 1, 2018 data are from Experiment 2, and 2020 data are from Experiment 3

We next classified subjects into three political groups according to their self-reported political identification. We classified subjects as “Left” when they had selected any of the “left” options (*n* = 92), “Center” when they had selected the “center” option (*n* = 54), and “Right” when they had selected any of the “right” options (*n* = 57). In the analyses that follow, we found similar patterns of results when treating political identification as a continuous variable; our classifications here are for the sake of simplicity of interpretation.

**Fig. 2:**
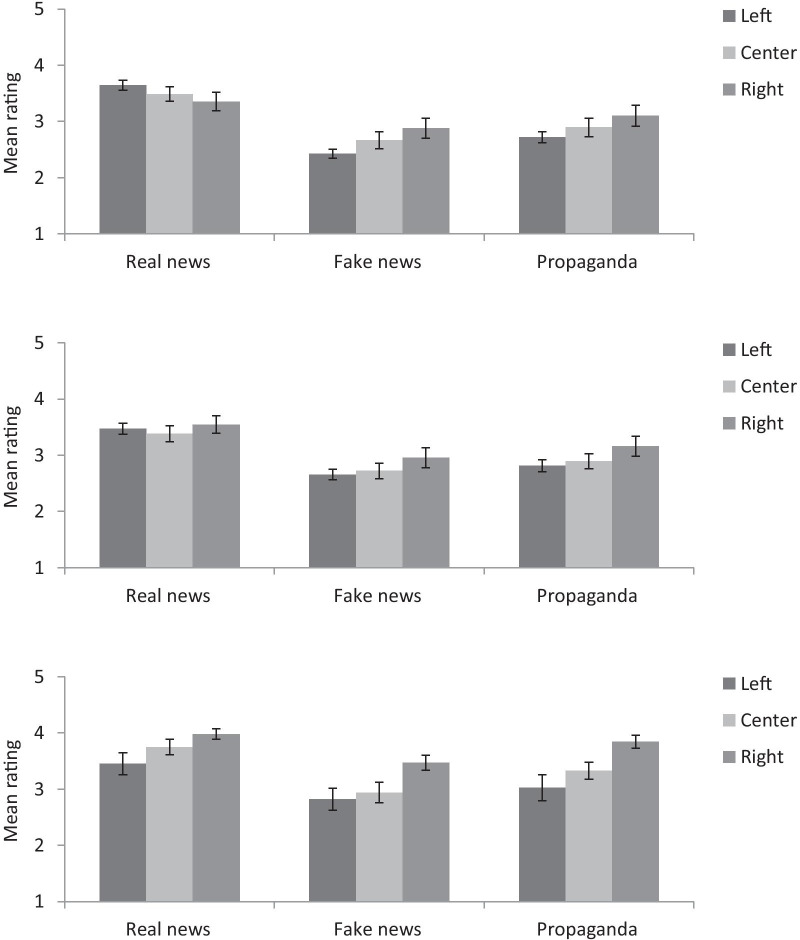
Average Real news, Fake news, and Propaganda ratings—split by Political identification. Top panel: 2017 data. Middle panel: 2018 data. Bottom panel: 2020 data. Error bars represent 95% confidence intervals of cell means

Before turning to our primary questions, we wondered how people’s ratings varied according to political identification, irrespective of news source. To the extent that conservatives believe claims that the mainstream media is “fake news,” we might expect people on the right to have higher overall ratings of fake news and propaganda than their counterparts on the left. Conversely, we might expect people on the left to have higher overall ratings of real news than their counterparts on the right. We display the three averaged ratings—split by political identification—in the top panel of Fig. 2. As the figure shows, our predictions were correct. One-way analyses of variance (ANOVAs) on each of the three averaged ratings, treating Political Identification as a between-subjects factor with three levels (Left, Center, Right), were statistically significant: Real news *F*(2, 200) = 5.87, *p* = 0.003, *η*^2^ = 0.06; Fake news *F*(2, 200) = 13.20, *p* < 0.001, *η*^2^ = 0.12; Propaganda *F*(2, 200) = 7.80, *p* < 0.001, *η*^2^ = 0.07.^2^ Follow-up Tukey comparisons showed that people who identified left gave higher real news ratings than people who identified right (*M*_diff_ = 0.29, 95% CI [0.09, 0.49], *t*(147) = 3.38, *p* = 0.003, Cohen’s *d* = 0.492); lower fake news ratings than people who identified right (*M*_diff_ = 0.45, 95% CI [0.24, 0.66], *t*(147) = 5.09, *p* < 0.001, *d* = 0.771) and center (*M*_diff_ = 0.23, 95% CI [0.02, 0.44], *t*(144) = 2.59, *p* = 0.028, *d* = 0.400); and lower propaganda ratings than people who identified right (*M*_diff_ = 0.39, 95% CI [0.15, 0.62], *t*(147) = 3.94, *p* < 0.001, *d* = 0.663). Together, these results suggest that—compared to their liberal counterparts—conservatives generally believe that the news sources included in this study provide less real news, more fake news, and more propaganda.

We now turn to our primary questions. First, to what extent does political affiliation affect which specific news sources people consider real news, fake news, or propaganda? To answer that question, we ran two-way ANOVAs on each of the three rating types, treating Political Identification as a between-subjects factor with three levels (Left, Center, Right) and News Source as a within-subject factor with 42 levels (i.e., Table 1).^3^ These analyses showed that the influence of political identification on subjects’ ratings differed across the news sources. All three ANOVAs produced statistically significant interactions: Real news *F*(2, 82) = 6.88, *p* < 0.001, *η*^2^ = 0.05; Fake news *F*(2, 82) = 7.03, *p* < 0.001, *η*^2^ = 0.05; Propaganda *F*(2, 82) = 6.48, *p* < 0.001, *η*^2^ = 0.05.

Because follow-up comparisons would prove unwieldy, we instead adopted an exploratory approach to investigate these interactions. Specifically, for each of the 42 news sources, we calculated the mean differences between political identification groups (Left, Center, Right) for each of the three ratings subjects made (Real, Fake, Propaganda). We then ordered these data to highlight the largest differences. We display the 5 largest differences for each rating type in Table 3. As the table shows, many of the same news sources that liberals rated more highly as real news were rated more highly as fake news and propaganda by conservatives. In addition, each of these differences exceeded a value of one—representing an entire category shift up or down the 5-point rating scale.

**Table 3:** Maximum news source rating differences across political identification

Rating type	News Source	*M*_Diff_	95% CI	Direction
2017				
Real news	Breitbart	1.19	[0.73, 1.65]	Right > Left
	The New York Times	1.07	[0.68, 1.46]	Left > Right
	NPR	1.07	[0.67, 1.47]	Left > Right
	The Washington Post	1.05	[0.66, 1.44]	Left > Right
	MSNBC	1.03	[0.64, 1.43]	Left > Right
Fake news	The New York Times	1.36	[0.95, 1.78]	Right > Left
	CNN	1.32	[0.84, 1.79]	Right > Left
	MSNBC	1.24	[0.80, 1.67]	Right > Left
	The Washington Post	1.21	[0.78, 1.64]	Right > Left
	NPR	1.15	[0.74, 1.57]	Right > Left
Propaganda	The New York Times	1.28	[0.81, 1.75]	Right > Left
	Fox News	1.26	[0.79, 1.73]	Left > Right
	NPR	1.12	[0.65, 1.59]	Right > Left
	CNN	1.06	[0.55, 1.57]	Right > Left
	MSNBC	1.04	[0.52, 1.56]	Right > Left
2018				
Real news	Fox News	1.40	[0.93, 1.87]	Right > Left
	The Drudge Report	0.95	[0.55, 1.36]	Right > Left
	Fox News	0.88	[0.41, 1.35]	Center > Left
	Infowars	0.79	[0.38, 1.20]	Right > Left
	Breitbart	0.77	[0.33, 1.20]	Right > Left
Fake news	Fox News	1.11	[0.63, 1.59]	Left > Right
	Fox News	1.02	[0.54, 1.50]	Left > Center
	MSNBC	0.89	[0.43, 1.35]	Right > Left
	CNN	0.83	[0.34, 1.32]	Right > Left
	Occupy Democrats	0.82	[0.46, 1.19]	Right > Left
Propaganda	Fox News	1.02	[0.54, 1.50]	Left > Right
	CNN	0.94	[0.45, 1.43]	Right > Left
	Fox News	0.91	[0.43, 1.39]	Left > Center
	San Francisco Chronicle	0.76	[0.38, 1.14]	Right > Left
	The Washington Post	0.73	[0.28, 1.19]	Right > Left
2020				
Real news	The Daily Caller	1.07	[0.66, 1.48]	Right > Left
	The Blaze	1.02	[0.63, 1.41]	Right > Left
	The Drudge Report	1.01	[0.56, 1.45]	Right > Left
	David Avocado Wolfe	0.98	[0.52, 1.43]	Right > Left
	The Daily Mail	0.96	[0.56, 1.37]	Right > Left

Top 5 rating differences only

#### Beliefs about “fake news”

Recall our second primary question: To what extent does political affiliation affect how people interpret the term “fake news”? To answer that question, we analyzed the responses subjects gave when asked what “fake news” and “propaganda” mean. We analyzed only those responses in which subjects provided a meaningful response (95%, *n* = 192). We calculated the proportion of subjects whose responses: (1) indicated that “fake news” and “propaganda” were similar; (2) indicated that “fake news” and “propaganda” were different; (3) provided a shared definition that applied to both terms; and (4) provided a separate definition for each term.^4^ These categories were not mutually exclusive. Two raters developed operational definitions of these response characteristics before independently assessing responses, resolving discrepancies via discussion. Table 4 displays these data. As the table shows, the proportions of subjects whose responses included these characteristics were similar across political identification, barring one exception: Chi-square analyses revealed that people who identified left were more likely to provide separate definitions for the terms (80%) than people who identified right (65%), χ^*2*^(1, *N* = 149) = 6.37, *p* = 0.012, Odds Ratio (OR) = 2.58, 95% CI [1.23, 5.42], all other *p* values > 0.261. This finding suggests liberals believe fake news and propaganda are more distinct than conservatives. Consistent with this idea, proportionally fewer liberals gave a definition that applied to both terms (18%) than conservatives (28%), although this difference was not statistically significant (*p* = 0.261). For the interested reader, the Additional file 1 presents additional exploratory analyses examining subjects’ definitions.

**Table 4:** Proportion of subjects in each political identification group whose responses to the question about what fake news and propaganda mean included certain characteristics

Characteristic	Left	Center	Right
2017			
Indicated similar	0.16	0.22	0.17
Indicated different	0.03	0.04	0.02
Shared definition	0.18	0.26	0.28
Separate definitions	0.80	0.69	0.65
2018			
Indicated similar	0.18	0.06	0.17
Indicated different	0.03	0.00	0.10
Shared definition	0.30	0.40	0.32
Separate definitions	0.73	0.58	0.64
2020			
Indicated similar	0.05	0.20	0.20
Indicated different	0.05	0.20	0.14
Shared definition	0.19	0.20	0.22
Separate definitions	0.52	0.44	0.38

We collected these data in March of 2017. Given the tumultuous nature of the political climate, a question naturally arose: How consistent are these patterns over time? Considering that the topic of “fake news” has remained prevalent, we might expect that people are increasingly familiar with claims about which specific news agencies are supposed reporters of fake news. One possibility, then, is that people’s beliefs about sources of real and fake news are becoming increasingly divided. To examine this issue and build on our initial data, we replicated Experiment 1 in April of 2018.

## Experiment 2

This experiment was pre-registered (https://aspredicted.org/v2i73.pdf).

### Method

#### Subjects

We recruited a total of 204 Amazon Mechanical Turk workers who live in the USA (125 women, 77 men, 2 unreported; *M*_age_ = 39 years, age range: 18–74 years). According to a sensitivity analysis, this sample size gives us adequate power to detect a small interaction effect by conventional standards (*f* = 0.06).

#### Design

We again manipulated News Source within subjects and assigned subjects to one of three Political Identification groups based on responses to a political identification question.

#### Materials and procedure

The materials and procedure were identical to Experiment 1.

### Results and discussion

#### Beliefs about news sources

As in Experiment 1, we first examined the extent to which the three rating types subjects made were related to each other, collapsed across news sources. Table 2 presents the Pearson correlations for these three measures and their associated 95% CIs. As the table shows, real news ratings were once again negatively associated with fake news ratings and propaganda ratings, and fake news ratings were once again positively associated with propaganda ratings. In general, the relationships were like those in Experiment 1, although the negative correlations were smaller in this sample.

We next classified subjects into three political groups (Left: *n* = 81; Center: *n* = 62; Right: *n* = 61). Before turning to our primary questions, we wondered how people’s ratings varied according to political identification, irrespective of news source. We display the three averaged ratings—split by political identification—in the middle panel of Fig. 2. As the figure shows, the results are both similar and different to our earlier sample. One-way analyses of variance (ANOVAs) were statistically significant for fake news and propaganda ratings, but not real news ratings: Real news *F*(2, 201) = 1.45, *p* = 0.237, *η*^2^ = 0.01; Fake news *F*(2, 201) = 5.34, *p* = 0.006, *η*^2^ = 0.05; Propaganda *F*(2, 201) = 6.94, *p* = 0.001, *η*^2^ = 0.06. Follow-up Tukey comparisons showed that those on the right gave higher fake news and propaganda ratings than those on the left (Fake news: *M*_diff_ = 0.30, 95% CI [0.08, 0.52], *t*(140) = 3.18, *p* = 0.005, *d* = 0.528; Propaganda: *M*_diff_ = 0.35, 95% CI [0.12, 0.58], *t*(140) = 3.64, *p* = 0.001, *d* = 0.617) and higher propaganda ratings than centrists (*M*_diff_ = 0.27, 95% CI [0.03, 0.51], *t*(121) = 2.63, *p* = 0.025, *d* = 0.474). Together, these results show that—compared to their liberal counterparts—conservatives still generally believe that the news sources used in this study provide more fake news and more propaganda. In contrast to the earlier sample, however, we did not find that conservatives believe the news sources provide less real news.

We now turn to our primary questions. First, to what extent does political affiliation affect which news sources people consider real news, fake news, or propaganda? To answer that question, we ran two-way ANOVAs on each of the three rating types, treating Political Identification as a between-subjects factor with three levels (Left, Center, Right) and News Source as a within-subject factor with 42 levels (i.e., Table 1). These analyses showed that the influence of political identification on subjects’ ratings differed across the news sources. All three ANOVAs produced statistically significant interactions: Real news *F*(2, 82) = 3.50, *p* < 0.001, *η*^2^ = 0.03; Fake news *F*(2, 82) = 3.56, *p* < 0.001, *η*^2^ = 0.03; Propaganda *F*(2, 82) = 3.56, *p* < 0.001, *η*^2^ = 0.03.

We adopted the approach from Experiment 1 to investigate these interactions, displaying the 5 largest differences for each rating type in Table 3. The table shows some consistency with Experiment 1, but also some differences. We again found that some of the same sources liberals rated more highly as real news were rated more highly as fake news and propaganda by conservatives. But the largest differences appeared in a few different sources in this sample. Moreover, the differences in general were reduced in magnitude; only 4 exceeded a value of 1, representing an entire category shift along the rating scale.

#### Beliefs about “fake news”

Recall our second primary question: To what extent does political affiliation affect how people interpret the term “fake news”? To answer that question, we analyzed the responses subjects gave when asked what “fake news” and “propaganda” mean. As in Experiment 1, we analyzed only those responses in which subjects offered a meaningful response (88%, *n* = 180). Table 4 displays these data. As the table shows, the proportions of subjects whose responses included the characteristics described in Experiment 1 were similar across political identification. Specifically, we did not replicate the finding from Experiment 1, wherein people who identified left were more likely to provide separate definitions for the terms than people who identified right, χ^*2*^(1, *N* = 127) = 1.08, *p* = 0.300, OR = 1.50, 95% CI [0.70, 3.22].

Considered together, the results of Experiments 1 and 2 are consistent, in that they show partisan distinctions with respect to which news agencies are considered real and fake news. The data from Experiment 2, however, suggest that people’s views about individual sources are malleable and that—contrary to what we hypothesized—the partisan divide may be shrinking. To what extent would such trends remain consistent? To answer that question, we ran a third experiment in late August of 2020 that replicated Experiments 1 and 2. We also took this opportunity to explore two potential explanations for any partisan differences.

The first explanation is that people rely on familiarity with a news source as a guide in determining the extent to which it provides real and fake news. We know from a growing body of research that familiarity increases the ease of information processing, which in turn leads to judgments of truth (Dechêne et al. 2010; Oppenheimer 2008). We might expect, then, that people judge more familiar news sources as real news, less familiar news sources as either fake news or propaganda, and that people on the left are familiar with different sources than people on the right. To test this idea, we included an exploratory measure of familiarity for each news source.

The second explanation is that partisan differences may be driven by differences in the tendency to think analytically. Recent research shows that this tendency helps people distinguish plausible and implausible news information, regardless of political ideology (Pennycook and Rand 2019). That finding suggests that what drives differences in beliefs about real and fake news is not necessarily a partisan bias—that is, a motivation to reason in favor of a particular ideology—but instead differences in the tendency to engage in effortful, analytic thinking. We might expect, then, that differences in beliefs about news sources themselves are similarly the result of differences in the tendency to think analytically. To test this idea, we included an exploratory measure of people’s tendency to engage in effortful reasoning.

## Experiment 3

This experiment was pre-registered (https://aspredicted.org/bs5sh.pdf).

### Method

#### Subjects

To boost precision, we aimed to recruit 300 Amazon Mechanical Turk workers who live in the USA. A total of 325 people participated, but 32 failed to complete the experiment. The resulting dataset comprises 293 people (103 women, 190 men; *M*_age_ = 36 years, age range: 21–69 years). According to a sensitivity analysis, this sample size gives us adequate power to detect a small interaction effect by conventional standards (*f* = 0.05).

#### Design

We again manipulated News Source within subjects and assigned subjects to one of three Political Identification groups based on responses to a political identification question.

#### Materials and procedure

The materials and procedure were identical to Experiments 1 and 2, except as follows.

We asked subjects to rate how familiar they were with each news source—presented in alphabetical order—on a scale from 1 (*Not at all familiar*) to 5 (*Very familiar*). This rating task followed immediately after the phase in which subjects provided real news, fake news, and propaganda ratings for the news sources.^5^

Subjects then completed a Cognitive Reflection Test (CRT) as a measure of analytic thinking (Frederick 2005). This test comprises 3 questions that tend to elicit different answers when thinking relatively effortlessly versus effortfully. For example, one question asks: “A bat and a ball cost $1.10. The bat costs $1.00 more than the ball. How much does the ball cost?” The intuitive answer is 10 cents, but a more analytic approach reveals the correct answer of 5 cents. For each of the three CRT questions, subjects provided a numeric response.

### Results and discussion

#### Beliefs about news sources

As in Experiments 1 and 2, we first examined the extent to which the three rating types subjects made were related to each other, collapsed across news sources. Table 2 presents the Pearson correlations for these three measures and their associated 95% CIs. As the table shows, real news ratings were positively associated with fake news and propaganda ratings, and fake news ratings were positively associated with propaganda ratings. In general, two of these three associations are markedly different from those reported in Experiments 1 and 2 and could reflect a shift over time in people’s beliefs about the type of information reported by our list of news sources.

We next classified subjects into three political groups (Left: *n* = 33; Center: *n* = 78; Right: *n* = 182). Before turning to our primary questions, we wondered how people’s ratings varied according to political identification, irrespective of news source. We display the three ratings—split by political identification—in the bottom panel of Fig. 2. As the figure shows, the results are both similar and different to our earlier samples. One-way ANOVAs were statistically significant for all three averaged ratings: Real news *F*(2, 292) = 11.19, *p* < 0.001, *η*^2^ = 0.07; Fake news *F*(2, 292) = 15.18, *p* < 0.001, *η*^2^ = 0.09; Propaganda *F*(2, 292) = 25.25, *p* < 0.001, *η*^2^ = 0.15. Follow-up Tukey comparisons showed that conservatives gave higher real news ratings than liberals or centrists (Right-Left *M*_diff_ = 0.53, 95% CI [0.24, 0.81], *t*(213) = 4.37, *p* < 0.001, *d* = 0.715; Right-Center *M*_diff_ = 0.23, 95% CI [0.03, 0.43], *t*(258) = 2.70, *p* = 0.020, *d* = 0.315); higher fake news ratings than liberals or centrists (Right-Left *M*_diff_ = 0.65, 95% CI [0.27, 1.04], *t*(213) = 4.01, *p* < 0.001, *d* = 0.886; Right-Center *M*_diff_ = 0.53, 95% CI [0.26, 0.80], *t*(258) = 4.55, *p* < 0.001, *d* = 0.719); and higher propaganda ratings than liberals or centrists (Right-Left *M*_diff_ = 0.82, 95% CI [0.49, 1.15], *t*(213) = 5.87, *p* < 0.001, *d* = 1.110; Right-Center *M*_diff_ = 0.52, 95% CI [0.28, 0.75], *t*(258) = 5.17, *p* < 0.001, *d* = 0.700). Together, these results are consistent with Experiments 1 and 2 in suggesting that—compared to their liberal and centrist counterparts—conservatives generally believe that the sources used in this study provide more fake news and more propaganda. What appears to have changed over time is that conservatives now also believe that these sources report more real news.

We now turn to our primary questions. First, to what extent does political affiliation affect which news sources people consider real news, fake news, or propaganda? To answer that question, we ran two-way ANOVAs on each of the three rating types, treating Political Identification as a between-subjects factor with three levels (Left, Center, Right) and News Source as a within-subject factor with 42 levels (i.e., Table 1). These analyses showed that the influence of political identification on subjects’ ratings differed across the sources—but only for real news ratings: *F*(2, 82) = 1.57, *p* < 0.001, *η*^2^ = 0.01, all other interaction effect *p* values > 0.280.

We again adopted the approach from Experiments 1 and 2 to investigate this interaction, displaying the largest 5 differences in Table 3. The table shows a partisan divide, with conservatives rating these news sources more highly as sources of real news than liberals. In addition, these differences are close to or greater than a value of 1, representing an entire category shift up or down the rating scale. Perhaps of note is that in comparison with the 2017 and 2018 data, none of these news sources are traditional, mainstream agencies.

#### Beliefs about “fake news”

Recall again our second primary question: To what extent does political identification affect how people interpret the term “fake news”? To answer that question, we again analyzed the responses subjects gave when asked what fake news and propaganda mean. We analyzed only those responses in which subjects offered a definition for either term (55%, *n* = 162). Note that the proportion of subjects who provided such definitions was lower than in Experiments 1 (95%) and 2 (88%). Upon closer examination, we found that several subjects had likely pasted definitions from an Internet search. In an exploratory analysis, we found a statistically significant difference in the likelihood that participants provided a pasted definition, based on Political Identification, χ^*2*^ (2, *N* = 162) = 7.66, *p* = 0.022. Specifically, conservatives (23%) were more likely than centrists (6%) to provide a pasted definition, χ^*2*^ (1, *N* = 138) = 7.29, *p* = 0.007, OR = 4.57, 95% CI [1.29, 16.20], all other *p* values > 0.256. Liberals fell between these extremes, with 13% providing a pasted definition. Because we were interested in subjects’ own definitions, we excluded these suspicious responses from analysis (*n* = 27).

We then followed the same analytic procedure as in Experiments 1 and 2. Table 4 displays these data. As the table shows, the proportions of subjects whose responses included the characteristics described in Experiment 1 were similar across political identification. Specifically, we did not replicate the finding from Experiment 1, wherein people who identified left were more likely to provide separate definitions for the terms than people who identified right, χ^*2*^ (1, *N* = 90) = 1.42, *p* = 0.233, all other *p* values > 0.063.

#### Additional exploratory analyses

We now turn to our additional exploratory analyses specific to this experiment. First, we examine the extent to which people’s reported familiarity with our news sources varies according to their political identification. Liberals and conservatives may be familiar with different sources, and we know that familiarity can act as a guide in determining what is true (Alter and Oppenheimer 2009). To examine this idea, we ran a two-way ANOVA on familiarity, treating Political Identification as a between-subjects factor with three levels (Left, Center, Right) and News Source as a within-subject factor with 42 levels (i.e., Table 1). This analysis showed that the influence of political identification on subjects’ familiarity ratings differed across the sources: *F*(2, 82) = 2.11, *p* < 0.001, *η*^2^ = 0.01. Closer inspection revealed that conservatives reported higher familiarity than liberals for most news sources, with centrists falling in-between (*F*s range 6.62—23.27, *M*_Right-Left_ range 0.62—1.39, all *p* values < 0.002). The exceptions—that is, where familiarity ratings were not meaningfully different across political identification—were the media giants: The BBC, CNN, Fox News, Google News, The Guardian, The New York Post, The New York Times, The Wall Street Journal, The Washington Post, Yahoo News, and CBS News.

We also predicted that familiarity with our news sources would be positively associated with real news ratings and negatively associated with fake news ratings. To test this idea, we calculated—for each news source—correlations between familiarity and real news ratings, and familiarity and fake news ratings. In line with our prediction, we found that familiarity was positively associated with real news ratings across all news sources: maximum *r*_Real_(292) = 0.48, 95% CI [0.39, 0.57]; minimum *r*_Real_(292) = 0.15, 95% CI [0.04, 0.26]. But in contrast with what we predicted, we found that familiarity was also positively associated with fake news ratings, for two out of every three news sources: maximum *r*_Fake_(292) = 0.34, 95% CI [0.23, 0.44]; minimum *r*_Fake_(292) = 0.12, 95% CI [0.01, 0.23]. Only one of the remaining 14 sources—CNN—was negatively correlated, *r*_*Fake*_(292) = -0.15, 95% CI [-0.26, -0.03]; all other CIs crossed zero. Taken together, these exploratory results, while tentative, might suggest that familiarity with a news source leads to a bias in which people agree with any claim about that source.

Next, we examined how the tendency to think analytically influences people’s interpretations of news sources. We know from related work that people who think more analytically—regardless of political affiliation—are better able to discern real news headlines from fake news headlines (Pennycook and Rand 2019). We might therefore expect that some of our observed differences relate to the ability to think analytically. We calculated a CRT performance score for each subject ranging from 0 to 3, according to whether each subject gave correct (+ 1) or incorrect (+ 0) answers to the three CRT questions. Most of the sample answered zero questions correctly (67%, *n* = 196), 18% answered one correctly (*n* = 53), 11% answered two correctly (*n* = 31), and the remaining 4% answered all questions correctly (*n* = 13). We then compared CRT scores across political identification and found that liberals scored higher than centrists and conservatives, *F*(2, 292) = 4.52, *p* = 0.012, *η*^2^ = 0.03; Left-Center *M*_Diff_ = 0.49, 95% CI [0.08, 0.90], *p* = 0.015, *d* = 0.58; Left–Right *M*_Diff_ = 0.46, 95% CI [0.08, 0.83], *p* = 0.013, *d* = 0.54.

Next, we explored how the tendency to think analytically affected real news, fake news, and propaganda ratings of the various news sources. Specifically, we ran repeated-measures analyses of covariance (RM-ANCOVAs) on each rating type, treating news source as a within-subject factor and CRT score as a continuous covariate. For real and fake news ratings, we found that the influence of analytic thinking interacted with news sources: *F*_Real_(41, 251) = 2.60, *p* < 0.001, *η*^2^ = 0.01; *F*_Fake_(41, 251) = 1.81, *p* = 0.003, *η*^2^ = 0.003. Closer inspection showed that higher scores on the CRT led to lower real news ratings for less reputable news sources, such as Infowars and Occupy Democrats: the 14 statistically significant *B*s ranged from -0.29 to -0.14. Higher CRT scores also led to lower fake news ratings for highly reputable news sources, such as Reuters and the Associated Press: the 12 statistically significant *B*s ranged from -0.28 to -0.16.^6^ For propaganda ratings, however, we found only a main effect of the tendency to think analytically: *F*_Propaganda_(1, 292) = 9.80, *p* = 0.002, *η*^2^ = 0.03, *B* = -0.17. Together, these patterns of results suggest that the tendency to engage in critical thinking helps people differentiate between high- and low-quality news sources. Given the exploratory nature of these analyses, the skew of the CRT scores, and the relatively small pool of subjects who identified “Left,” we encourage cautious interpretation of these findings.

## General discussion

In this investigation into the “fake news” phenomenon, we wanted to examine what people believe constitutes fake news. We also wanted to examine which specific news sources people believe real news and fake news come from, and whether such beliefs relate to political affiliation. We asked people to rate the extent to which a variety of news sources report real news, fake news, and propaganda. We also asked people to tell us what they think these terms mean. The key results of interest were in line with our predictions: The ratings people gave depended on the relationship between their political affiliation and a news source. In general, news sources rated more highly as real news by liberals were rated more highly as fake news and propaganda by conservatives, and vice versa. But both things cannot be true. The results are consistent with an explanation in which people’s political motivations influence their reasoning strategies (Epley and Gilovich 2016; Kunda 1990). Put another way, people’s beliefs regarding the news might reflect a desirability bias (Tappin et al. 2017). These findings are potentially worrying. If people’s beliefs about the credibility of news sources are determined in part by political affiliation, then unwarranted labeling of reputable news agencies as fake news by political groups could exacerbate media distrust among that group’s constituents.

We also found that conservatives viewed our list of news agencies, on average, more as sources of fake news and propaganda than liberals. That finding fits with prior work showing a general distrust of news media among conservatives (Lee 2010). But one counter-explanation for this pattern of results is that our list might be skewed, consisting more of sources traditionally associated with the left. Considering the range of our sources, we suspect this explanation is unlikely, or at least insufficient. It would also be difficult to square that explanation with the finding from Experiment 3, in which conservatives also viewed our list of news agencies, on average, more as sources of real news than liberals.

We found some tentative evidence that people’s beliefs about specific news sources are changing—at least in some respects. Although many of the findings were consistent across our samples, there were three key differences. First, the correlations between real news on the one hand, and fake news and propaganda on the other, shifted from highly negative in 2017, to moderately negative in 2018, to slightly positive in 2020. Second, we found that conservatives viewed the list of news agencies, on average, less as sources of real news than liberals in 2017—but this difference was absent in 2018 and reversed in 2020. Third, the specific news agencies rated most different across political affiliation changed somewhat in each sample, and in the most recent sample we found no evidence of meaningful political affiliation differences for fake news and propaganda ratings. Taken together, this collection of results hints at a potential bridging of the divide across the political spectrum with respect to beliefs about media reporting. Additionally, the results suggest that people’s classifications of news sources as real, fake, or propaganda are malleable. We make these claims only tentatively, however, given the nature of our sampling.

Recall that in our third experiment, we sought to explore two potential explanations for any observed partisan differences. First: familiarity. More specifically, that people trust news sources they know, distrust those they do not, and that liberals and conservatives are familiar with different news sources. Although we cannot rule out this explanation, we suspect it is unlikely for a few reasons. First, most of our sources are well-known, due to how we constructed the list. Second, when we examined the data, we found no clear differences between these more well-known sources and the additional eight, potentially less well-known sources we added to the list. Third, ratings of source familiarity in Experiment 3 were positively correlated with beliefs that news sources report both real and fake news. Moreover, data from a related, ongoing project suggest that differences in trust across news sources are not driven by familiarity (Michael and Sanson 2021). Taken together, these findings suggest that source familiarity on its own is not a strong determinant of beliefs about the sorts of news those sources provide.

The second potential explanation we explored was that differences in beliefs about news sources might reflect differences in the tendency to think analytically. Specifically, that it is not partisan motivations that drive judgments about sources of real and fake news, but instead differences in the tendency to engage in critical thought. We found tentative support for this idea: Stronger analytic thinking led to lower real news ratings of dubious sources, and lower fake news of reputable sources—although the magnitude of this influence varied across sources. These results dovetail with research showing that analytic thinking is a useful predictor of the ability to sort fact from fiction in news headlines (Pennycook and Rand 2019). The data also suggest—in line with other recent work—that motivated reasoning, in some contexts, is an insufficient explanation for how people form beliefs and preferences (Druckman and McGrath 2019; Pennycook and Rand 2019).

One limitation of this work is that we classified people into political groups based on a single self-report measure. This simplistic classification limits the inferences we can draw. Although the measure has face validity, it arguably lacks depth and may not have good construct validity. Future work incorporating established measures that tap into constructs underpinning political beliefs could provide more useful information about the potential mechanisms at play (e.g., Right Wing Authoritarianism from Altemeyer 1981; or Social Dominance Orientation from Pratto et al. 1994, but see the target article by Hibbing et al. 2014 and ensuing peer commentary in the June 2014 issue of Behavioral and Brain Sciences for more nuanced discussion).

Another limitation is that the data are subjective. More specifically, our subjects made judgments about sparse information: We do not have an objective measure of the extent to which our news sources provide real or fake news. Thus, we cannot determine who is more “correct” in their beliefs about these news sources. This subjectivity stands in contrast to the recent work wherein subjects made judgments about news headlines—information that could be more reliably checked for veracity (Pennycook and Rand 2019). But this subjectivity raises interesting questions for future research. For example, our findings suggest that the same news information, when attributed to different sources, would be interpreted differently depending on people’s political affiliation (Michael and Sanson 2021). That hypothesis, if true, is consistent with a motivated reasoning explanation and is reminiscent of the persuasive effects of the perceived credibility of a source (Petty and Cacioppo 1986). It would also extend research investigating how the presence or absence of source information affects news interpretations (Pennycook and Rand 2019).

A further limitation relates to the source of our subject pool. Concerns have been raised about the quality of data from Mechanical Turk, including a lack of diversity and participation driven by monetary desires. But surprisingly, studies on Mechanical Turk have been shown to produce high-quality data on par with laboratory results across several tasks (Buhrmester et al. 2011; Casler et al. 2013). Nonetheless, we also know that most tasks are completed by a relatively small pool of subjects who may communicate with one another (Peer et al. 2017). Because we had no control over subjects’ communications and did not limit participation to naïve workers, we cannot rule out the possibility that these confounds are present in our data. In addition, we noted an increase in what appears to be satisficing behavior in our most recent sample (Hamby and Taylor 2016). One potential solution to these issues would be to collect additional data from only naïve Mechanical Turk subjects, or from another subject pool—like a traditional university sample or an alternative crowdsourcing market. If the results converge across these samples, we could be confident that such confounds do not meaningfully distort the data.

Our findings have implications for the reporting of news information and people’s ensuing beliefs. Considering that we found people’s political affiliation influences their beliefs about the reporting of real and fake news from various news sources, one implication is that people’s beliefs about news information are driven more by the source of that information than attempts to sort fact from fiction. Specifically, people might discount information as “fake news” when it comes from a source that they believe is politically incongruent. This implication leads to a prediction: People on either side of the political spectrum may be more easily deceived by misinformation when it comes from a source that aligns with their political ideology. If that prediction is correct, it would fit with research showing that people are more susceptible to misinformation from credible sources (Dodd and Bradshaw 1980; Echterhoff et al. 2005; French et al. 2011). Note, however, that recent research adds nuance to this body of work, showing that distrusted sources can make plausible news seem less plausible, and that distrust blossoms when purportedly fake news is discovered to be real (Dias et al. 2020; Wang and Huang 2020).

Another implication stems from the strong positive correlations between fake news and propaganda ratings across all three experiments. Those findings suggest that people think about fake news and propaganda in somewhat similar ways, so it is worth exploring in future research the extent to which people find these terms interchangeable. Preliminary research suggests that the meanings of these two terms overlap, but are distinguishable, and that political affiliation might influence how the terms are defined (Breaux and Dauphinet 2021). For example, when asked to describe examples of fake news, people’s reports range from propaganda, to poor journalism, to outright false news—and even include misleading advertising (Nielsen and Graves 2017).

The findings also have potential applications. The data suggest that recent movements aimed at helping people to distinguish fake news from real news are not only necessary, but that these movements need to take care in how they construct their material with respect to source information. Specifically, the movements stand to benefit from acknowledging that political affiliation feeds into skepticism—or lack thereof—when encountering news information from different sources. Relatedly, recent work suggests another worrying trend affecting people’s interpretations of news information: an increase in sensationalist reporting from reputable news agencies (Spillane et al. 2020).

The “fake news” phenomenon occupies a unique moment in history. While the popularity of the phrase may dwindle over time, it remains to be seen what consequences this labeling of information will ultimately have on people’s beliefs regarding the news (Additional file 1).

## Supplementary Information

**Additional file 1**. Supplementary materials including additional exploratory analyses.

## Data Availability

Data are available from https://osf.io/x7jnu/.

## Footnotes

1 We found that this variable explained only trivial additional variance, and thus we will not discuss it further.

2 In each experiment, we first ran a multivariate analysis of variance (MANOVA), resulting in a statistically significant main effect of Political Identification (all *p* values < .001).

3 In each experiment, we first ran a MANOVA, resulting in a statistically significant interaction between Political Identification and News Source (all *p* values < .001).

4 We additionally coded responses according to: (1) whether or not a definition for “fake news” included the word “propaganda” and vice versa; (2) whether or not the subject defined only one term; (3) whether or not the subject indicated uncertainty about the meaning of either term. However, we had no reason to predict systematic differences across political affiliation for these response categories, so they were not included in our analyses.

5 We also asked subjects who they voted for in the 2016 US presidential election, with the option to select between Donald Trump, Hillary Clinton, or Other. This question appeared immediately after subjects reported what the terms “fake news” and “propaganda” mean. We report additional exploratory analyses investigating the influence of subjects’ age and voting behavior in the supplementary materials.

6 For real news ratings, the statistically significant sources were: Addicting Info, AOL, The Atlantic, Breitbart, The Daily Caller, The Daily Mail, David Avocado Wolfe, The Drudge Report, Infowars, The New York Daily News, Occupy Democrats, Russia Today, The Blaze, and US Uncut. For fake news ratings, the statistically significant sources were: The Associated Press, The BBC, The Boston Globe, The Chicago Tribune, The Economist, The Intercept, NPR, The New York Times, Reuters, USA Today, The Wall Street Journal, Washington Post.
