# Supplementary material for: The relationship between political affiliation and beliefs about sources of “fake news”
Source: Cogn Res Princ Implic. 2021 Feb 12;6:6. doi: 10.1186/s41235-021-00278-1 (PMC7880518; doi:10.1186/s41235-021-00278-1)
Supplement: Supplementary file 1 — Additional file 1. Supplementary materials including additional exploratory analyses. [file 41235_2021_278_MOESM1_ESM.docx]

The relationship between political affiliation and beliefs about sources of “Fake News”

Robert B. Michael, Brooke O. Breaux

University of Louisiana at Lafayette

Author Note

Address correspondence to:

Robert B. Michael

Assistant Professor

Department of Psychology

University of Louisiana at Lafayette

PO Box 43644

Lafayette, LA, 70504-3644

[robert.michael@louisiana.edu](mailto:robert.michael@louisiana.edu)

+1 (337) 482 6585

**Supplementary Materials**

# Experiment 1

***Beliefs about “Fake News”***

We examined the content of the definitions subjects provided. The same two raters independently partitioned responses into three categories: parts defining fake news, parts defining propaganda, and parts defining neither. Discrepancies were again resolved via discussion, resulting in data from ten additional subjects being excluded due to providing inadequate definitions. We then used Linguistic Inquiry and Word Count (LIWC2015) software to analyze those parts defining fake news.^1^ LIWC2015 is a language analysis tool that calculates how often words associated with different psychologically relevant categories—or “dictionaries”—appear in a target text (Pennebaker et al., 2015).

We ran one-way ANOVAs on the average percentage of words subjects produced that are found in 13 LIWC2015 dictionaries, treating Political Identification as a between-subjects factor with three levels (Left, Center, Right; see Table 1). We selected dictionaries that focus on words from affective and cognitive categories, based on meta-analytic work showing differences in these factors across political affiliation (Jost et al., 2003). Overall, we found no statistically significant differences, barring one exception: People in the center were slightly less likely than those on the left (*M*_diff_ = 1.86, 95% CI [0.34, 3.39], *d* = 0.43) or right (*M*_diff_ = 1.71, 95% CI [0.00, 0.87], *d* = 0.40) to use words found in the Insight dictionary, *F*(2, 178) = 3.18, *p* = .044, η^2^ = .03. Some examples of words from this dictionary subjects produced are: “believe,” “evidence,” “information,” “persuade,” and “prove.” Although speculative, we suspect that this difference reflects less serious consideration on the part of centrists when defining “fake news.”

# Experiment 2

***Beliefs about “Fake News”***

We examined the content of subjects’ definitions, following the same analytic procedure as in Experiment 1 (see Table 1). Across the 13 LIWC2015 dictionaries, we found no statistically significant differences according to Political identification (all *p* values > .150). Specifically, we did not replicate the finding from Experiment 1, wherein centrists were less likely than liberals or conservatives to use words found in the Insight dictionary.

# Experiment 3

***Beliefs about “Fake News”***

We examined the content of subjects’ definitions, following the same analytic procedure as in Experiments 1 and 2 (see Table 1). We found a statistically significant effect of Political Identification on the use of words reflecting Cognitive processes, *F*(2, 97) = 4.73, *p* = .011, ƞ*^2^* = .089. In particular, words found within the Causation dictionary, *F*(2, 97) = 5.96, *p* = .004, ƞ*^2^* = .109, and words found within the Tentative dictionary, *F*(2, 97) = 3.23, *p* = .044, ƞ*^2^* = .062. Closer inspection showed that liberals used proportionally more language related to Cognitive processes than conservatives and centrists (Left-Right *M*_diff_ = 12.40, 95% CI [0.63, 24.17], *t*(67) = 2.09, *p* = .039, *d* = 0.553; Left-Center *M*_diff_ = 19.71, 95% CI [6.99, 32.43], *t*(47) = 3.08, *p* = .003, *d* = 0.879). More specifically, liberals used proportionally more “causation” words than conservatives and centrists (Left-Right *M*_diff_ = 9.98, 95% CI [3.86, 16.10], *t*(67) = 3.24, *p* = .002, *d* = 0.846; Left-Center *M*_diff_ = 10.30, 95% CI [3.69, 16.92], *t*(47) = 3.09, *p* = .003, *d* = 0.873). Some examples of causation words subjects produced are “create,” “intent,” “purpose,” “manipulate,” and “mislead.” Liberals and conservatives both used proportionally more “tentative” words than centrists (Left-Center *M*_diff_ = 5.29, 95% CI [0.61, 9.98], *t*(47) = 2.24, *p* = .027, *d* = 0.650; Right-Center *M*_diff_ = 3.85, 95% CI [0.25, 7.45], *t*(80) = 2.12, *p* = .036, *d* = 0.473). Some examples of tentative words subjects produced are “opinion,” “dubious,” “try,” “usually,” and “sometimes.”

***Additional exploratory analyses***

**Age.** We explored how subjects’ reported age influenced interpretations of our news sources. We modified our ANOVA analyses on average real news, fake news, and propaganda ratings into ANCOVAs, treating Age as a continuous covariate. We found no evidence that age meaningfully altered the pattern of results (all *p* values > .336).

**Voting behavior.** We explored how subjects’ voting behavior in 2016 influenced interpretations of our news sources. We modified our ANOVA analyses on average real news, fake news, and propaganda ratings, replacing the Political Identification factor with subjects’ 2016 Presidential vote (Trump, Clinton, or Other). For real news ratings, we found no statistically significant influence of 2016 Presidential vote, *F*(2, 290) = 0.80, *p* = .450. For fake news ratings, we found a statistically significant influence of 2016 Presidential vote, *F*(2, 290) = 9.96, *p* < .001, η^2^ = .06. Follow-up Tukey comparisons revealed that 2016 Trump voters believed our sources reported more fake news than Clinton and Other voters (Trump-Clinton *M*_diff_ = 0.44, 95% CI [0.14, 0.74], *t*(281) = 3.44, *p* = .002, *d* = 0.562; Trump-Other *M*_diff_ = 0.89, 95% CI [0.23, 1.56], *t*(231) = 3.16, *p* = .005, *d* = 1.148). For propaganda ratings, we found a statistically significant influence of 2016 Presidential vote, *F*(2, 290) = 7.46, *p* = .001, η^2^ = .05. Follow-up Tukey comparisons revealed that 2016 Trump voters believed our sources reported more propaganda than Clinton and Other voters (Trump-Clinton *M*_diff_ = 0.37, 95% CI [0.10, 0.63], *t*(281) = 3.24, *p* = .004, *d* = 0.472; Trump-Other *M*_diff_ = 0.60, 95% CI [0.01, 1.19], *t*(231) = 2.39, *p* = .045, *d* = 0.774).

References

Jost, J. T., Glaser, J., Kruglanski, A. W., & Sulloway, F. J. (2003). Political conservatism as motivated social cognition. *Psychological Bulletin, 129*, 339-375. doi:10.1037/0033-2909.129.3.339

Pennebaker, J. W., Boyd, R. L., Jordan, K., & Blackburn, K. (2015). *The development and*

*psychometric properties of LIWC2015*. Austin, TX: University of Texas at Austin.

Footnotes

We focused this analysis on fake news definitions because fake news and propaganda were highly correlated, and our primary interest was to examine how people define fake news across the political spectrum.

Tables

Table 1

*Percentage of words in subjects’ definitions of fake news that were found in LIWC dictionaries, separated by Political Identification.*

| Dictionary | Left | Center | Right |
| --- | --- | --- | --- |
| 2017 |  |  |  |
| *Affective processes* | *7.94* | *9.64* | *6.68* |
| Positive emotion | 3.33 | 3.88 | 3.74 |
| Negative emotion | 4.53 | 5.76 | 2.93 |
| Anxiety | 0.25 | 0.34 | 0.00 |
| Anger | 2.88 | 3.40 | 0.97 |
| Sadness | 0.07 | 0.52 | 0.20 |
| *Cognitive processes* | *27.72* | *24.18* | *29.6* |
| Insight | 2.62 | 0.75 | 2.46 |
| Causation | 6.16 | 5.16 | 8.69 |
| Discrepancy | 0.34 | 1.19 | 0.53 |
| Tentative | 7.04 | 5.32 | 5.41 |
| Certainty | 7.60 | 7.12 | 6.46 |
| Differentiation | 7.52 | 7.10 | 8.64 |
| 2018 |  |  |  |
| *Affective processes* | *8.14* | *9.03* | *9.84* |
| Positive emotion | 4.26 | 4.97 | 4.26 |
| Negative emotion | 3.74 | 4.06 | 3.98 |
| Anxiety | 0.27 | 0.00 | 0.00 |
| Anger | 0.62 | 2.68 | 0.26 |
| Sadness | 0.04 | 0.00 | 0.00 |
| *Cognitive processes* | *25.20* | *31.83* | *29.46* |
| Insight | 3.34 | 3.21 | 2.95 |
| Causation | 4.58 | 6.52 | 5.61 |
| Discrepancy | 0.28 | 0.48 | 0.10 |
| Tentative | 4.86 | 6.66 | 5.71 |
| Certainty | 5.22 | 6.42 | 9.27 |
| Differentiation | 7.78 | 11.55 | 9.35 |
| 2020 |  |  |  |
| *Affective processes* | *5.65* | *11.06* | *15.23* |
| Positive emotion | 2.65 | 3.21 | 7.94 |
| Negative emotion | 3.00 | 7.85 | 7.30 |
| Anxiety | 0.00 | 0.00 | 0.00 |
| Anger | 2.58 | 0.00 | 1.96 |
| Sadness | 0.00 | 0.00 | 0.09 |
| *Cognitive processes* | *32.84* | *13.13* | *20.44* |
| Insight | 1.85 | 2.33 | 1.42 |
| Causation | 12.21 | 1.91 | 2.24 |
| Discrepancy | 0.52 | 0.36 | 0.30 |
| Tentative | 6.37 | 1.08 | 4.93 |
| Certainty | 5.25 | 3.07 | 3.72 |
| Differentiation | 9.59 | 5.68 | 10.12 |
